# EXO1/P53/SREBP1 axis-regulated lipid metabolism promotes prostate cancer progression

**DOI:** 10.1186/s12967-023-04822-z

**Published:** 2024-01-26

**Authors:** Zefeng Wang, Zheng Chao, Qi Wang, Fan Zou, Tianbao Song, Lizhe Xu, Jinzhuo Ning, Fan Cheng

**Affiliations:** 1 Department of Urology, Renmin Hospital of Wuhan University, Wuhan, 430060 China; 2 Department of Urology, Tongji Hospital, Tongji Medical College, Huazhong University of Science and Technology, Wuhan, Hubei China; 3 Department of Urology, Union Hospital, Tongji Medical College, Huazhong University of Science and Technology, Wuhan, Hubei China

**Keywords:** Prostate cancer, Lipid metabolism, EXO1, P53, Biomarker

## Abstract

Prostate cancer (PCa) is one of the most common malignant tumors affecting the male genitourinary system. However, there is currently a lack of effective treatments for patients with advanced prostate cancer, which significantly impacts men’s overall health. Exonuclease 1 (EXO1), a protein with mismatch repair and recombination functions, has been found to play a vital role in various diseases. In our study, we discovered that EXO1 acts as a novel biomarker of PCa, which promotes prostate cancer progression by regulating lipid metabolism reprogramming in prostate cancer cells. Mechanistically, EXO1 promotes the expression of SREBP1 by inhibiting the P53 signaling pathway. In summary, our findings suggest that EXO1 regulated intracellular lipid reprogramming through the P53/SREBP1 axis, thus promoting PCa progression. The result could potentially lead to new insights and therapeutic targets for diagnosing and treating PCa.

**Supplementary Information:**

The online version contains supplementary material available at 10.1186/s12967-023-04822-z.

## Introduction

Prostate cancer (PCa) is a malignant genitourinary tumor and the second leading cause of cancer-related deaths in men globally. A previous study estimated that approximately 1.4 million new cases of PCa and 375,000 deaths occur annually worldwide [1]. Several risk factors contribute to PCa, including age, family history of tumors, lifestyle, environmental factors, and certain genetic mutations. Biochemical recurrence is observed in about one-third of patients following radical prostatectomy, and a significant number of these patients progress to desmoplasia-resistant [2]. The median survival of patients with desmoplasia PCa ranges from 9 to 30 months [3].

Exonuclease 1(EXO1) is a gene that encodes a protein with an exonuclease, and the RNase H enzyme plays a key role in mismatch repair and double-strand break repair [4]. EXO1 has been linked to various diseases including Aicardi-Gouteres syndrome [5]. Alternative splicing of the EXO1 gene produces two isoforms in humans, EXO1a and EXO1b. Moreover a -48-amino-acid truncation of the C-terminal region of EXO1a impairs its DNA excision activity. Several studies have shown a strong association between EXO1 and the prognosis of malignant tumors [6, 7], including PCa [8]. However, the exact mechanism by which EXO1 promotes PCa progression remains unclear.

P53 signaling is activated in response to stresses, which is essential for the survival of normal cells, and protecting themselves against tumorigenesis [9]. However, the P53 protein is encoded by the TP53 gene, which is mutated in most tumors, primarily in the DNA-binging region. These mutations result in a loss of functions required for tumor inhibition or even a gain of functions required for tumor progression [10]. P53 is frequently lost in aggressive PCa with neuroendocrine differentiation resulting in weakened effects of androgen receptor inhibitors and enhanced PCa cell proliferation. [11, 12]. Moreover, some studies found that P53 plays multiple roles in lipid metabolism regulation [13, 14].

In this study, we aimed to investigate whether EXO1 could serve as a prognostic factor for PCa using weighted gene co-expression network analysis (WGCNA). We found that EXO1 promoted PCa proliferation, and metastasis as well as lipid accumulation and disease progression. Mechanistically, EXO1 enhanced the expression of SREBP1 by inhibiting P53 signaling.

## Methods

### Dataset

Data related to the TCGA-PRAD project were obtained through the UCSC Xena Browser (https://xenabrowser.net/) [15]. GSE6919 from the GEO database (https://www.ncbi.nlm.nih.gov/geo/) [16]was used in this study [17, 18].

### Bioinformatics analysis

Detailed methods and principles have been reported in previous studies [19–21]. Briefly, expression and clinical trait data for the TCGA-PRAD project were downloaded from its official website. We used the “limma” package to obtain differential genes (adj. *P* < *0.05*, |logFC|> 1), and the differential genes were subsequently used in weighted co-expression network analysis with a soft threshold set to 4. The differential genes were categorized into 9 modules based on their expression patterns. Receiver operating characteristic (ROC) curves were generated to assess the diagnostic potential of EXO1 expression in PCa tissue. According to EXO1 expression, prostate cancer patients were categorized into two groups (high-expression group and low-expression group). The disease-free survival (DFS) of the two groups was expressed by Kaplan–Meier curves. Additionally, gene set enrichment analysis (GSEA) [22, 23] was conducted using the GSEA software (version 4.1.0, USA) to explore the enrichment of gene sets based on the expression data from the two groups mentioned.

### Prostate cancer tissues and cell lines

20 pairs of PCa tissues and normal tissues were acquired from patients at the Department of Urology, Renmin Hospital of Wuhan University. None of the patients received adjuvant therapy before surgery. By Wuhan University's Human Research Ethics Committee regulations, we conducted this study. According to the Helsinki Declaration, all procedures were followed in our research. To extract RNA, we utilized the Trizol reagent extraction kit (Cat: CW0580S, CWBIO, China) and used 1000 ng of RNA for cDNA synthesis (Cat: RK20429, Abclonal, China). The resulting cDNA was diluted 1:10 for quantitative PCR (qPCR). For qPCR, the cDNAs were combined with SYBR green PCR Master Mix (Cat: RK21203, Abclonal, China) and gene-specific primers listed in Additional file 6: Table S1. The qRT-PCR was performed on a LightCycler­^®^ 480 Instrument II from Roche. The obtained values were then normalized to the expression of the housekeeping gene β-actin. To extract protein from the samples, triplicate wells were lysed in chilled RIPA buffer (Cat: P0013C, Beyotime, China) containing complete protease inhibitors (Cat: HY-K0010, MedChemExpress, America) and Phostop phosphatase inhibitors (Cat: HY-K0021, MedChemExpression, America). The protein concentration of the lysates was quantified using the BCA protein quantification kit (Cat: P0010S, Beyotime, China). For SDS-PAGE (Cat: PG112, Epizyme, China) and western blotting analyses, the protein samples were suspended in Laemmli buffer and sonicated 15 times for 30 s with intermittent breaks before being loaded onto a gel. The samples were boiled and used for western blot analysis after the addition of β-mercaptoethanol and bromophenol blue. The membranes were blocked in EpiZyme fast-blocking buffer and incubated overnight at 4 °C with the primary antibodies in the blocking buffer containing 0.2% Tween-20. The primary antibodies used were EXO1 (Cat: 16253-1-AP, Proteintech, China) and ACTB (Cat: AC004, Abclonal, China). For secondary antibody treatment, the membranes were either blocked in fast blocking buffer (Cat: PS108P, EpiZyme, China) at room temperature for 10–15 min or incubated directly in the diluent-blocking buffer containing 0.2% Tween-20 and 0.01% SDS for 1 h at room temperature. The membranes were imaged using fluorescence on a Biorad Imager and processed using Adobe Photoshop CC 2018. To analyze gene expression, RNAs and protein samples were extracted from 10 paired tissue samples. Quantitative real-time polymerase chain reaction (qRT-PCR) was performed to analyze RNA samples, while immunoblotting tests were conducted for protein analysis. Meanwhile, four pairs of samples were analyzed via immunohistochemistry. For cell-based experiments, we used the normal prostate cell line RWPE1 and PCa cell lines PC3, DU145, and LNCaP, which were purchased from the American Type Culture Collection (ATCC). The medium was prepared by adding 10% fetal bovine serum (Cat: C2910-0500, BioMed, China) to 1640 medium (Cat: PM150110, Pricella, China) with 1% 100 × penicillin (Cat: PB180120, Pricella, China). Cells were incubated at 37 ℃ with 5% CO_2_ under controlled conditions.

### Immunoblotting test (IBT)

Cells or ground tissue were lysed using RIPA lysis buffer (Cat: P0013C, Beyotime, China) supplemented with proteasome inhibitors (Cat: ST507, Beyotime, China) for 30 min, during which time they were sonicated three times 10 s each. bicinchoninic acid (Cat: P0010S, Beyotime, Cina) was added to the lysate for 30 min at 37 °C. Then, the protein concentration of the lysate was measured and mixed with 5X loading buffer (Cat: BL502B, biosharp, China). After boiling at 100 °C for 10 min, the mixture was used for electrophoresis for 70 min, followed by transfer onto 0.45um PVDF membrane (Cat: IPVH00010, Merck, German). After that, 5% skimmed milk (Cat: GC310001, Servicebio, China) was soaked close to the PVDF membrane for 90 min. Next, the PVDF membrane was incubated with primary and secondary antibodies. After being washed with TBST (Cat: G0001, Servicebio, China) three times, the PVDF membrane was subjected to Electrochemiluminescence (ECL). The detailed information on primary and secondary antibodies employed in this study is presented in Additional file 1: Table S1.

### RNA extraction and qRT-PCR

The detailed experimental procedures were carried out as described in a study [24]. Trizol reagent (Cat: CW0580S, CWBIO, China) and RNApure Tissue & Cell Kit (Cat: CW0560, CWBIO, China) were used to extract RNA following the manufacturer’s protocol. 1 ng RNA was used for reverse reaction and then used for qPCR reaction with 2 × Universal SYBR Green Fast qPCR Mix (Cat: RK21203, Abclonal, China). ACTB served as an internal control. The primer sequences [25, 26] employed in this study are presented in Additional file 6: Table S1.

### IHC assay

Fresh tissues were immersed in 4% paraformaldehyde (Cat: G1101, Servicebio, China) and subsequently embedded into paraffin. Tissue sections were deparaffinized in water and subsequently washed well using PBS (Cat: G420202, Servicebio, China). Subsequently underwent antigen repair with hydrogen peroxide (Cat: 10011208, Sinopharm, China) and BSA (Cat: 4240GR005, BioFroxx, German) closure, placed in primary antibody working solution, and incubated overnight at 4 °C, followed by incubation of secondary antibody (Cat: ab205804, Abcam, Britain) as well as color development. Finally, structurally intact areas were selected for microscopic photographs. The detailed information of the primary employed in this study is presented in Additional file 6: Table S1.

### Plasmids, lentiviral construction, and transfection

Plasmids overexpressing or knocking down EXO1 (Cat: GS1-23040034, Genecreate, China), siRNA targeting P53, and their corresponding negative controls were constructed in Genecreate (Wuhan, China). PCa cells were collected after being transfected for 48 h using lipofectamine 3000 (Invitrogen, Carlsbad, CA) reagent, following the manufacturer's instructions. Lentivirus for the knockdown of EXO1 was constructed in Genechem (Shanghai, China) with a lentivirus backbone.. plasmids for the expression of SREBP1 were constructed in Genechem (Cat: GV272, China). With a lentivirus backbone, the sequence information of siRNA and shRNA is as follows.

**Table:** 

Target	Sequence
sh1-EXO1	GGAUGUACUUUACCUUCUAUU
sh2-EXO1	GAAGTAGAGAGATCTAGAA
si P53-1	CCGCGCCATGGCCATCTACA
si P53-2	GCUUCGAGAUGUUCCGAGA

### Hyperlipidemic cell model

PCa cells were cultured in 6-well plates. Oleic acid (OA) was formulated in two concentrations of 100uM, and 200uM according to the dissolution protocol of the manual (Cat: HY-N1446, MedChemExpress, America). PCa cells were divided into three groups, which were pulled into corresponding concentrations of OA, the control was BSA. After culturing cells for 48 h, transwell nesting (Cat: 3422, Corning, America) was used to evaluate the abilities of migration and invasion. Cell numbers of the image results were counted by Image J (National Institutes of Health, America), The statistical graphs were made up of GraphPad Prism 9 (America).

### Cell proliferation assays and EdU assays

Cells were collected by centrifugation and then resuspended using the medium at a density maintained at 10^4^ per ml of medium. Subsequently, 200 μl of culture medium was pulled into the wells of the 96-well culture plate. Cell proliferation assays were performed using CCK-8 (MedChemExpress, USA) and measured at 0, 24, 48 h, and 72 h according to the method provided by the manufacturer. Subsequently, the optical density at 450 nm (OD450) of the cells was measured and recorded using GraphPad Prism software over four days to reflect the proliferative ability of the cells. EdU-555 assay kit (C0075S, Beyotime) was applied for EdU assays. Following the protocol, when the cells reached 50% confluency, they were fixed and stained with the appropriate reagents. The recommended reaction time of 2 h was used. A fluorescence microscope (Olympus, Japan) at 200 × magnification was used to capture images. Cell proliferative ability was assessed, and three times were repeated for each group.

### Cell migration and invasion assays

Briefly, PCa cells were starved using a serum-free medium (Cat: 8122122, Gibco, America) for 8 h, then digested and centrifuged to collect the cells, which were subsequently resuspended using a serum-free medium. We used transwell nesting (Cat: 3422, Corning, America) to assay the invasive and migratory capacity of cells. For migration experiments, 40,000 cells were added to each chamber. For invasion experiments, 80,000 cells were added to each well. The time was set to 24–36 h. A microscope (Olympus, Japan) at 200 × magnification was used to capture images. Each group was repeated three times.

### Wound healing assay

Cells from each group were cultured in 6-well plates. When the fusion of the cells was close to 100%, the same size wound was created using a sterile lance tip. The wounds were observed and photographed at 0 h and 48 h, respectively.

### Colony formation assays

Cells (1000 PCa cells) were grown in wells in 6-well plates and cultured under normal conditions for 12 days. To fix the colonies, 4% paraformaldehyde was added to plates in each group for 10 min. The crystal violet (G1014, Servicebio) was used for staining. Finally, the colonies in each group were photographed.

### Oil red O staining

When PCa cells fusion reached 30%-50%, they were fixed by 4% paraformaldehyde and stained using Oil Red O staining solution at room temperature for 1 h. After being washed three times using PBS and air-drying, the cells were photographed using a microscope at 400 × magnification.

### TG and THO measurements

Directed by the manufacturer's instruction, cells were cultured using 10 cm dishes and collected for lysis using 100 μl 2% TritonX-100 (Cat: BS084, Biosharp, China). Separate groups were established for blanks, calibration, and sample, each with four replicate wells. Then, the groups were subjected to a total cholesterol assay kit (Cat: A111-1-1, Njjcbio, China) or triglyceride assay kit (Cat: A110-1-1, Njjcbio, China) incubated in a 37℃ cell culture incubator for 10 min. Then, the signal values of each group were detected at 500 nm wavelength. Finally, the total cholesterol and cholesterol content of each group were calculated according to the following formula. T-CHO/TG (mmol/gprot) = [(A sample-A blank)/(A calibration—A blank)] * C calibration /Cpr (C calibration: the concentration of calibration, Cpr: the concentration of sample).

### Tumor formation assay

Animal experiments were approved by the Laboratory Animal Ethics Committee of Wuhan University. PC3 cell lines with stable knockdown of EXO1 were constructed using lentivirus. The above cells were digested centrifuged resuspended using PBS and injected subcutaneously into the axilla of Balb/c nude mice (aged 4 weeks, procured from Cyagen Corporation and housed under pathogen-free conditions at the Animal Biosafety Level 3 Laboratory of Wuhan University) using a syringe cell injection of 5 × 10^6^ cells per mouse. The nude mice were kept in an SPF environment for approximately 30 days. Tumor growth was measured every 5 days starting from day 10. The mice were euthanized after the experiment, and the tumors were surgically removed and photographed. They were then used for IHC and tissue oil red staining.

### Statistical analyses

Data from each group were presented as mean ± standard deviation (SD). Student's t-test or paired Student's t-test was used to test differences between groups. The relationship between EXO1 expression and clinical traits of PCa patients was tested by Pearson's χ^2^ test. GraphPad Prism (California, USA) was used for all the aforementioned analyses above. Univariate and multivariate Cox regression analyses were performed with SPSS 25.0. The significance level was set at *P* < 0.05. Three repetitions of each experiment were conducted. The hypothetical mechanistic draw was made by Figdraw. *P < 0.05; **P < 0.01; ***P < 0.001; ****P < 0.0001. Error bars indicate mean ± SEM.

## Results

### WGCNA identified the hub module for PCa

**Fig. 1:**
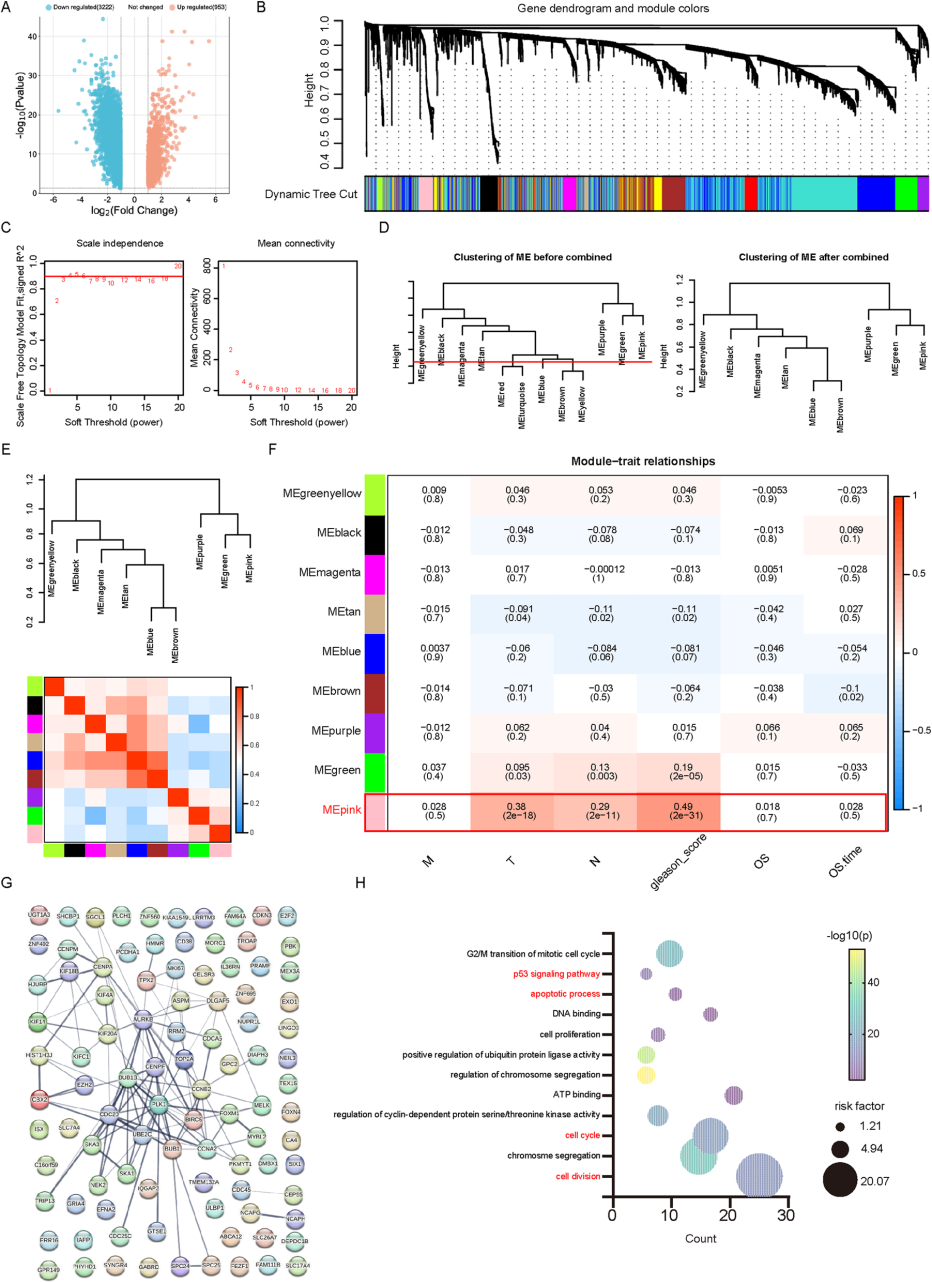
WGCNA analysis identified a hub module for PCa. **A** 4175 differential genes shown by volcano maps. **B** The gene dendrogram and module colors of DEGs are based on TOM. **C** Variation of scale-free topology and mean connectivity under different soft thresholds. **D** Clustering of modules before and after the merging. **E** The eigengene adjacency heatmap of 9 modules. **F** The relationships between nine modules and clinical traits. **G** The PPI network of genes in the pink module. **H** The GO and KEGG enrichment analysis of genes in the pink module

We obtained a total of 4175 differentially expressed genes (DEGs) from The Cancer Genome Atlas Prostate Adenocarcinoma (TCGA-PRAD) project meeting the criteria of |logFC|> 1, and adj* P* < 0.05. Among DEGs, 3222 were found to be lowly expressed genes, while 953 were highly expressed genes (Fig. 1A). We performed WGCNA to explore the expression patterns of these 4175 DEGs. Gene dendrogram and module colors were displayed (Fig. 1B). In this study, a scale-free network was determined with the soft threshold (β = 4) (Fig. 1C). Modules with similarity ≥ 0.75 were merged (Fig. 1D, E). Ultimately, nine modules were identified, and their relationships with clinical traits were demonstrated (Fig. 1F). Notably, the pink module exhibited the strongest association with clinical traits (T-stage: r = 0.38, *P* = 2e − 18; N stage: r = 0.29, *P* = 2e − 11; Gleason score: r = 0.49, *P* = 2e − 31). Therefore, the pink module, which contained 110 genes, was considered the hub module. Their protein interactions were revealed using the STRING database [27] (Fig. 1G). Gene Ontology (GO), and Kyoto Encyclopedia of Gene, and Genomes (KEGG) analyses [28, 29]showed that pink module genes have various functions in biological processes including cell cycle, apoptosis, and P53 signaling pathway (Fig. 1H).

### High EXO1 expression in PCa predicted poorer prognosis

**Fig. 2:**
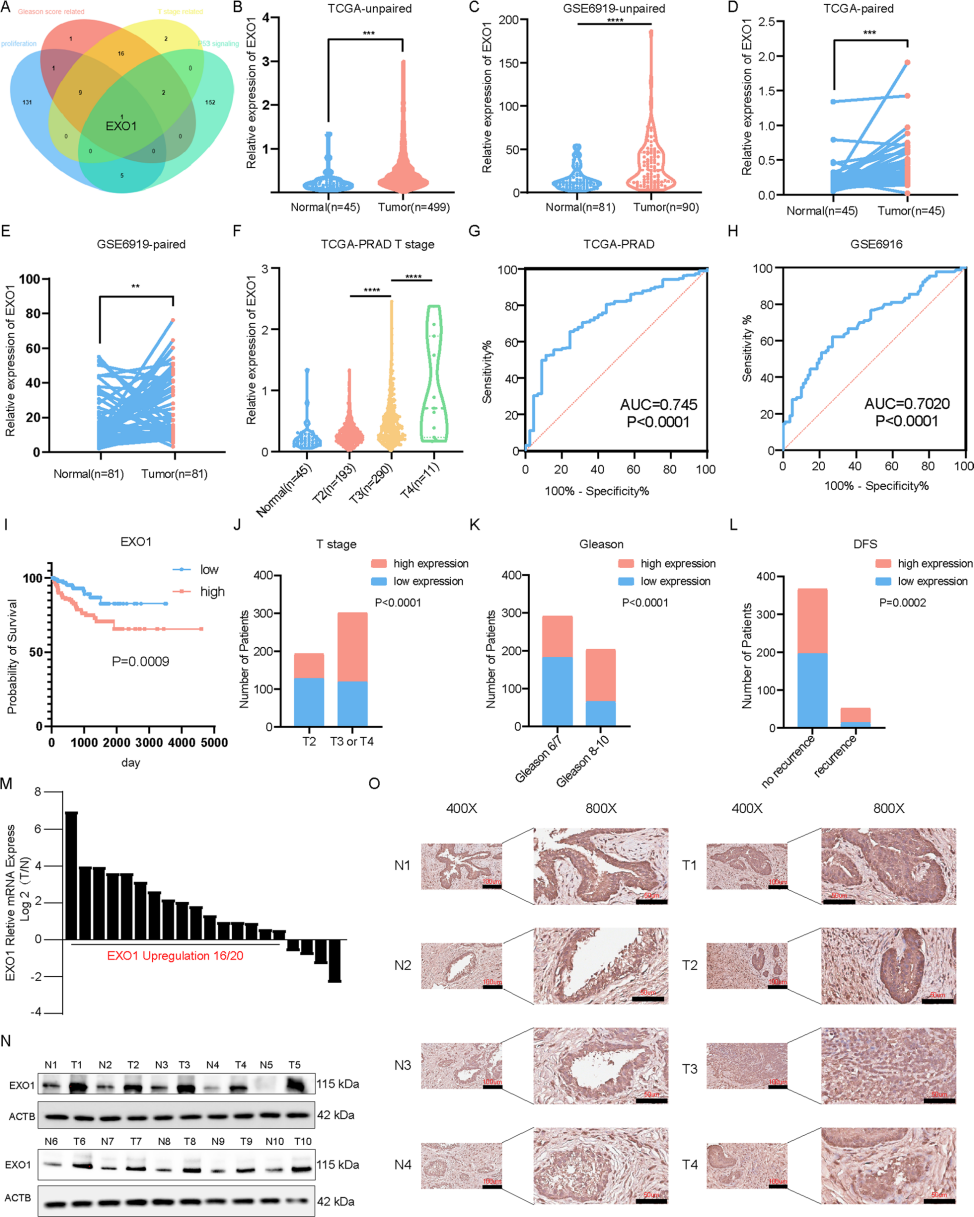
EXO1 was a good biomarker and highly expressed in PCa. **A** The top 30 genes most relevant to Gleason and T staging in each pink module were selected as gene sets. The genes associated with proliferation and P53 were also selected as the other two gene sets. These four gene sets were utilized to make a Wayne diagram. **B**–**E** Using the TCGA-PRAD project and the GSE6919 project, unpaired versus paired EXO1 expression was demonstrated. **F** Using the TCGA-PRAD project, EXO1 expression levels were demonstrated with different T stage. **G**–**H** Using the TCGA-PRAD project and the GSE6919 project, the ROC curves of EXO1 were displayed. **I** The DFS curve of EXO1 using TCGA-PRAD project. **J**–**L** A chi-square analysis was utilized to demonstrate the relationship between EXO1 expression levels and T stage, Gleason score, and recurrence. **M** The EXO1 relative RNA expression in PCa tissues. **N** The EXO1 protein expression of PCa tissues. **O** IHC results of EXO1 in PCa tissues

Thirty pink module genes that were closest to the T stage and Gleason scores were selected to form two datasets. The gene EXO1 was obtained after intersecting these two gene sets with the P53-signaling-related gene sets, and cell-cycle-related gene sets (Fig. 2A). Analysis of the TCGA-PRAD project and Gene Expression Omnibus (GEO) database revealed that EXO1 expression was higher in PCa tissue compared to normal tissue, and increased with elevated T-stage (Fig. 2B–F). ROC curves indicated that EXO1 had a good diagnostic value (Figs. 2G, H). The Kaplan–Meier curves indicated that high EXO1 expression predicted poor prognosis (Fig. 2I). By compiling clinical data from patients with high, and low EXO1 expression, the results of chi-square analysis demonstrated that EXO1 expression was related to PCa T-stage, Gleason score, and prognosis (Fig. 2J–L). Univariate and multivariate regression analyses demonstrated that EXO1 expression was a prognostic factor independent of TNM staging, and Gleason score (Table 1). Immunohistochemistry (IHC), RT qPCR, and western blotting (WB) were used to examine EXO1 expression in PCa and paracancerous tissues. The results showed that EXO1 RNA levels were higher in cancerous tissue than in paracancerous tissues in 16/20 patients. Meanwhile, EXO1 protein expression levels were higher in cancerous tissue than in paracancerous tissues in all patients. (Fig. 2M–N). The IHC results showed that EXO1 was significantly higher in cancerous tissues than in paracancerous tissues (Fig. 2O). To investigate the reasons for the high expression of EXO1 in PCa, we explored the copy number variation (CNV) of EXO1 and its promoter methylation changes in the TCGA-PRAD project through the GSCA database [30]. The results showed that the CNV of EXO1 is amplified in PCa, whereas its promoter methylation level was hypomethylated (Additional file 1: Fig. S1A, C). Further exploration revealed that the expression level of EXO1 was positively correlated with its CNV and negatively correlated with its promoter methylation level (Additional file 1: Fig. S1B, D). These findings that the high expression of EXO1 in PCa may be caused by the amplification in CNV and the low level of promoter methylation.

**Table 1:** Univariate and multivariate analyses of CENPA mRNA level and patient disease-free survival (DFS)

Variable	Univariate analysis			Multivariate analysis ^c^		
	HR ^a^	90%CI ^b^	P	HR ^a^	90%CI ^b^	P
Disease-free survival (n = 486)						
EXO1						
Low (n = 242)	0.297	0.173–0.510	0.0002	0.506	0.285–0.899	0.05
High (n = 244)						
Age						
< 60 (n = 197)	0.642	0.395–1.043	0.133	0.875	0.537–1.426	0.654
> 60 (n = 289)						
T stage						
T2 (n = 190)	0.244	0.144–0.412	0.0001	0.473	0.266–0.840	0.032
T3 or T4 (n = 296)						
Gleason score						
Gleason 6/7 (n = 288)	0.244	0.144–0.412	0.00001	0.473	0.266–0.840	0.032
Gleason 8/9/10 (n = 198)						

^a^ Hazard ratio, estimated from the Cox proportional hazard regression model

^b^ Confidence interval of the estimated HR

^c^ Multivariate models were adjusted for age, T stage, and Gleason score

### EXO1 promoted PCa proliferation and migration in vitro

**Fig. 3:**
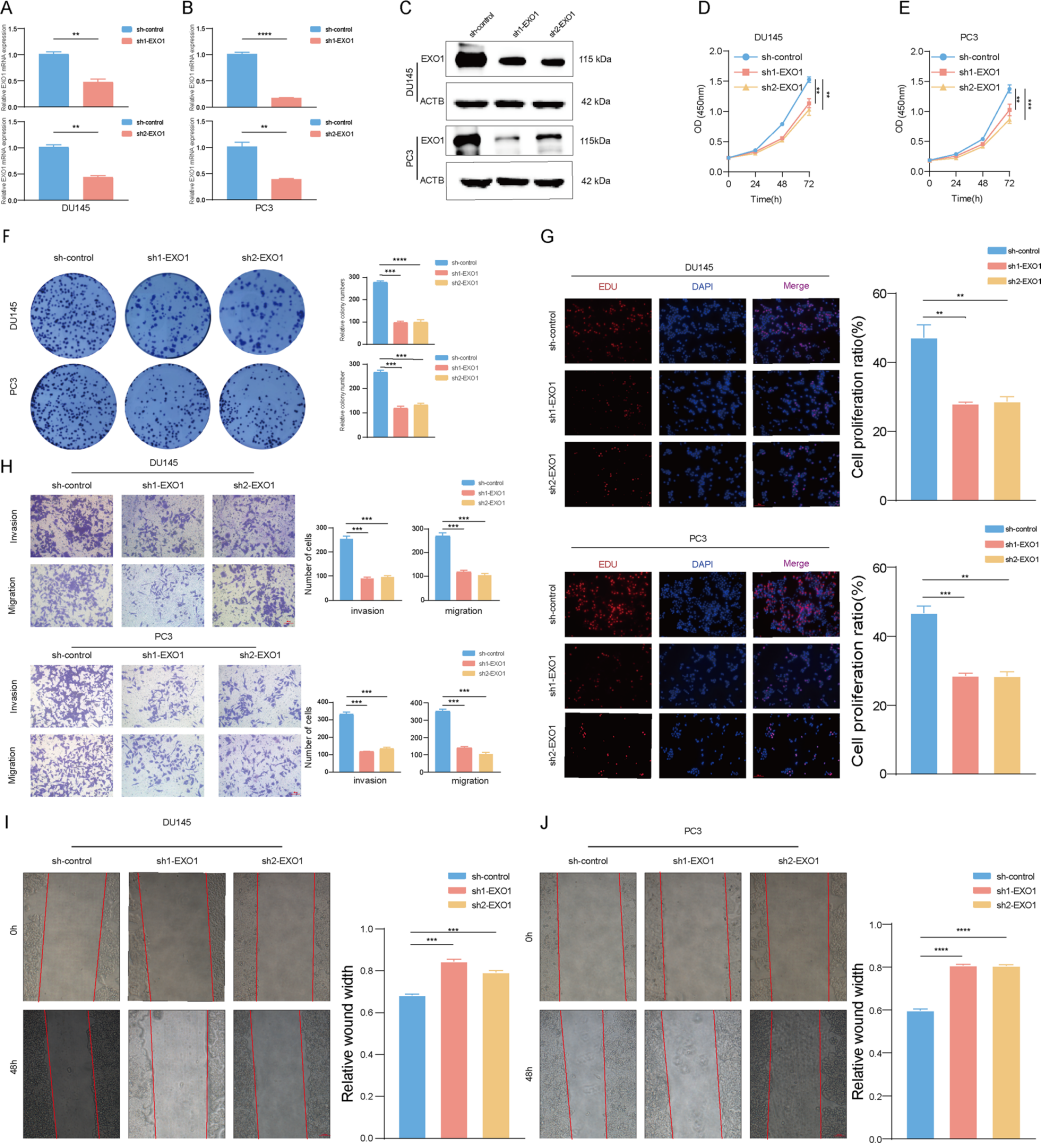
Knockdown of EXO1 inhibited PCa progression. **A**–**C** After the knockdown of EXO1 using lentivirus, the knockdown effect was verified using qPCR and Western blot. **D**, **E** After knocking down EXO1, using CCK-8 assays detected the prostate cancer cell's proliferation ability. **F** Cell proliferation was detected by knocking down EXO1 using colony formation assays. **G** Cell proliferation ability was detected by EdU assay after knocking down EXO1. **H**–**J** The metastatic ability of prostate cancer cells was detected using transwell and wound healing assays after knocking down EXO1

**Fig. 4:**
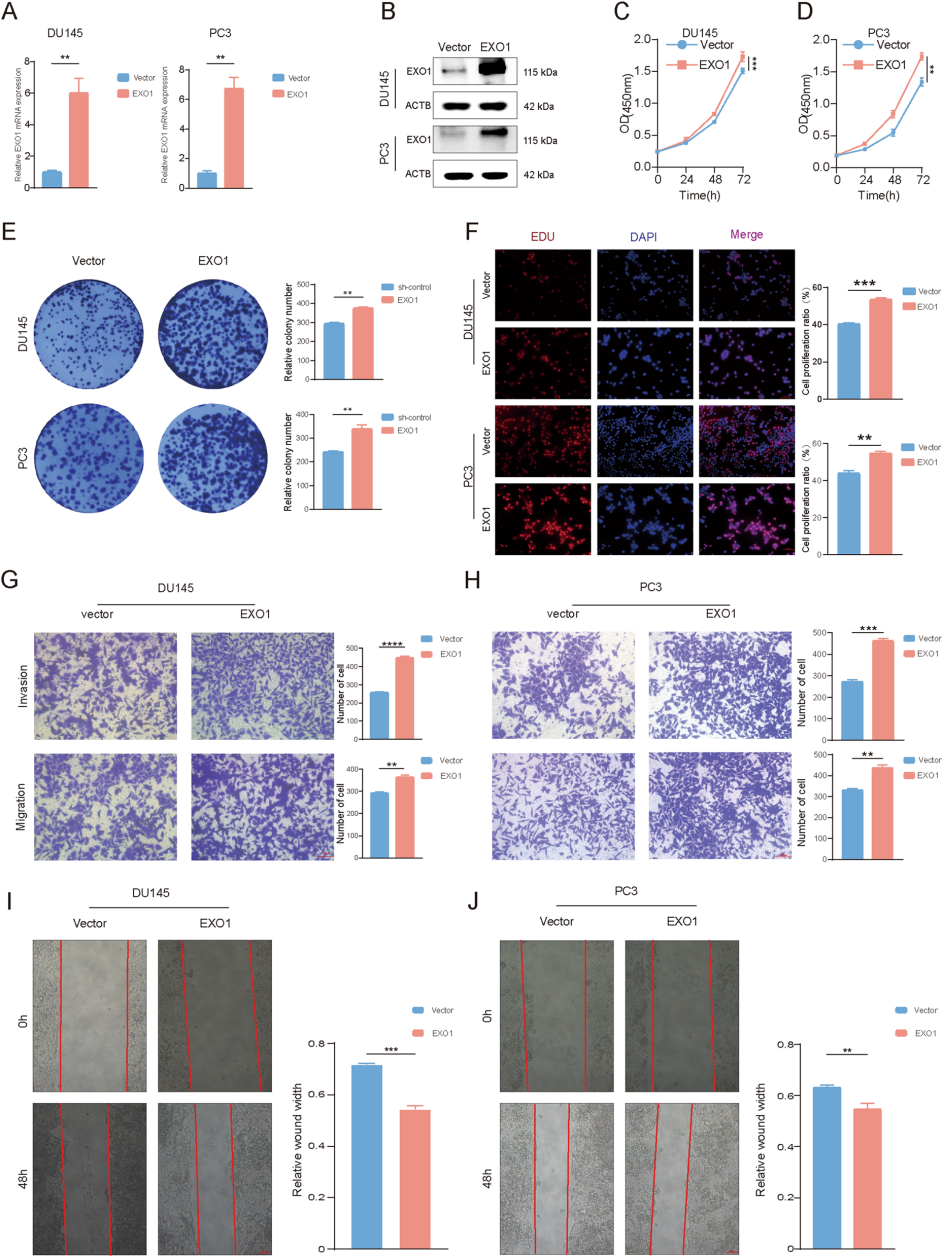
Overexpression of EXO1 promoted prostate cancer progression. **A**–**B** After overexpression of EXO1 using lentivirus, the effect was verified using qPCR and Western blot. **C**–**F** The proliferative capacity of cells after overexpression of EXO1 was assayed by CCK-8, colony formation, and EdU assay. **G**–**J** The metastatic ability of the cells was examined after overexpression of EXO1 using Transwell assay and wound healing assays

Drug sensitivity analysis [31] revealed that EXO1 was associated with sensitivity to a variety of antitumor drugs including vorinostat (Additional file 2: Fig. S2A, E). Immune infiltration analysis [32] showed that EXO1 promoted infiltration of activated CD4^+^positive T cells in multiple tumor types including prostate cancer (Additional file 2: Fig. S2F–I). Furthermore, prostate cancer patients were classified into four different immune subtypes (C1, C2, C3, and C4), and EXO1 expression was found to be the lowest in the C3 subtype (Additional file 2: Fig. S2J). As EXO1 is a promising prognostic biomarker for PCa, we investigated its role in PCa progression. EXO1 was stably knocked down in PCa cell lines (Fig. 3A–C). The results of Cell Counting Kit-8 (CCK-8), 5-ethynyl-2′-deoxyuridine (EdU) assay, and clone formation assays showed that the proliferative ability of PCa cells was effectively weakened by knocking down EXO1 (Fig. 3D–G). Transwell and wound healing assays showed that the metastatic ability of PCa cells was decreased after knocking down EXO1 (Fig. 3H–J). Conversely, over-expression of EXO1 significantly enhanced the cell proliferation, and migration abilities of PCa cells (Fig. 4A–J).

### EXO1 promoted lipid synthesis in PCa cells

**Fig. 5:**
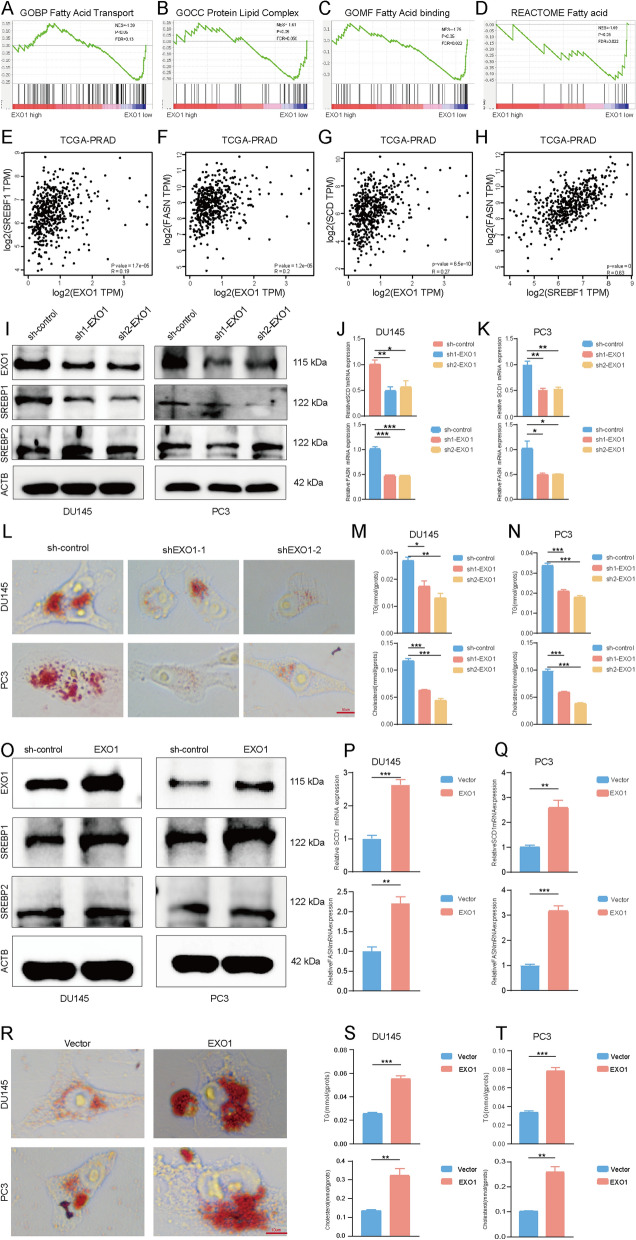
EXO1 inhibited lipid synthesis in prostate cancer. **A**–**D** GSEA analysis using the TCGA-PRAD project showed that EXO1 was associated with fatty acid transport and binding as well as protein-lipid complex formation. **E**–**G** Scatter plots were drawn using EXO1 expression as the horizontal coordinate and SREBP1, FASN, and SCD1 expression as the vertical coordinate. **H** Scatter plots with SREBP1 expression as the horizontal coordinate and FASN expression as the vertical coordinate. **I** Western blot detection of EXO1, SREBP1, and SREBP2 expression after knockdown of EXO1 in prostate cancer cells. **J**, **K** In prostate cancer cells, after the knockdown of EXO1, detect the expression of FASN and SCD1 by using qPCR. **L**–**N** In prostate cancer cells, after the knockdown of EXO1, lipid droplets were labeled using Oil Red O staining, and triglyceride and cholesterol levels were detected. **O** Overexpression of EXO1 in prostate cancer cells, and expression levels of EXO1, SREBP1, and SREBP2 were detected using Western Blot. **P**, **Q** After overexpression of EXO1, FASN, and SCD1 expression levels were detected in prostate cancer cells using qPCR. **R**–**T** After overexpression of EXO1 in prostate cancer cells, lipid droplets were labeled using Oil Red O staining, and triglyceride and cholesterol levels were detected

The results of Gene Set Enrichment Analysis (GSEA) indicated that EXO1 potentially regulated lipid metabolism (Fig. 5A–D). To find the relationship between PCa and lipid metabolism, different concentrations of OA were added to the culture medium. The results of CCK-8, transwell, and EdU assay suggested that high OA can promote the abilities of proliferation, migration, and invasion (Additional file 3: Fig. S3A–D). Among the key regulators of lipid resynthesis, the SREBP family, including SREBP1, and SREBP2, plays a significant role. In contrast to SREBP2, which regulated cholesterol resynthesis and uptake, SREBP1 was mainly responsible for fatty acid resynthesis, and regulated enzyme expression such as fatty acid synthase (FASN) and stearoyl-CoA desaturase 1 (SCD1). The results based on the Gene Expression Profiling Interactive Analysis (GEPIA) database [33] using the TCGA-PRAD project suggested that EXO1 expression in PCa was closely related to SREBP1, FASN, and SCD (Fig. 5E–G). SREBP1 was positively correlated with FASN expression in PCa (Fig. 5H). We found that SERBP1 expression was decreased, whereas SREBP2 expression remained relatively unchanged after EXO1 knockdown (Fig. 5I). Interestingly, FASN and SCD1 showed a consistent changing trend with SREBP1 (Fig. 5J–K). To validate the effects of EXO1 on lipid metabolism, we labeled intracellular lipid droplets using oil red O staining and examined the triglyceride (TG) and cholesterol levels in each group. Subsequently, the results of oil red O staining, TG, and cholesterol were consistent with the results of SREBP1 (Fig. 5L–N). By contrast, the expression level of SREBP1 increased after EXO1 overexpression (Fig. 5O). FASN and SCD1 followed the SREBP1 change (Fig. 5P–Q). We found that EXO1 promoted the TG, and cholesterol accumulation in PCa (Fig. 5R–T). Then we performed a rescue experiment to look for the relationship between EXO1 and SREBP1. In the plasmids-lentivirus-mediated EXO1 knockdown PCa cell lines, we conducted SREBP1 overexpression using plasmids and confirmed the upregulated expression of SREBP1 through WB analysis (Additional file 4: Fig. S4A). PCa cells proliferation, migration, and invasion become stronger than another group of knocking down EXO1 (Additional file 4: Fig. S4B, C, and H). The results of TG and cholesterol were consistent (Additional file 4: Fig. S4D–G). These results collectively suggested that EXO1 could upregulate SREBP1 to promote lipid synthesis.

### EXO1 inhibited P53 signaling in PCa

**Fig. 6:**
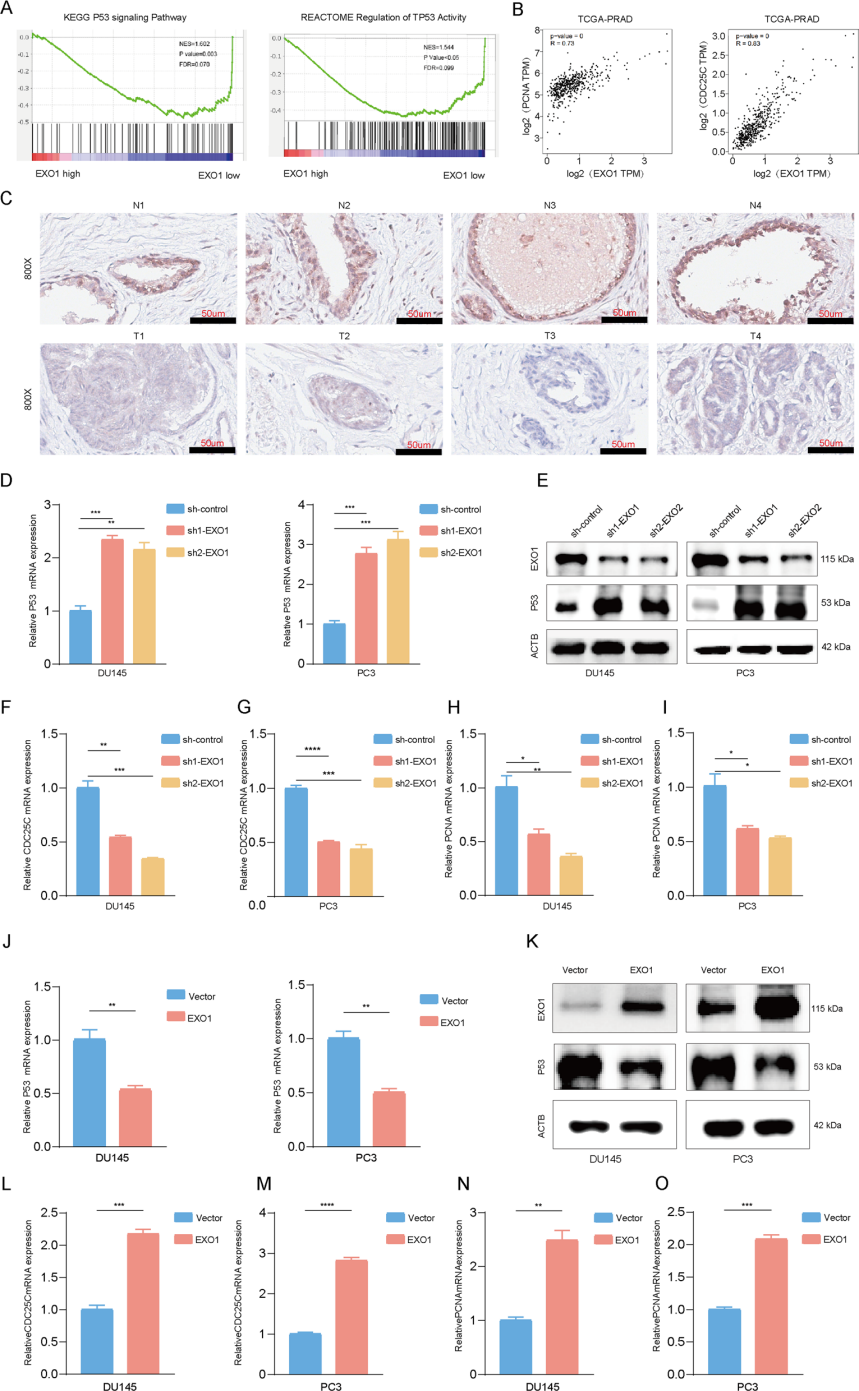
EXO1 inhibited P53 signaling in PCa. **A** GSEA analysis using the TCGA-PRAD project showed that EXO1 was associated with the activation and regulation of the P53 signal pathway **B** Scatter plots using the TCGA-PRAD program with EXO1 expression as the horizontal coordinate and PCNA and CDC25 expression as the vertical coordinate. **C** P53 Immunohistochemistry in 4 PCa of carcinoma and paraneoplastic. **D**, **E** Knockdown of EXO1 in prostate cancer cells and detection of P53 expression using Western blot and qPCR. **F**–**I** Expression of PCNA and CDC25 was detected by qPCR after the knockdown of EXO1. **J**, **K** After overexpression of EXO1 in prostate cancer cells, the expression level of P53 was detected using Western blot and qPCR. **L**–**O** After overexpression of EXO1, qPCR was used to detect the expression of PCNA and CDC25

Multigene GO and KEGG enrichment analysis of the pink module, where EXO1 was located, revealed an enrichment of the P53 signaling pathway (Fig. 1H). GSEA results further indicated that EXO1 was a potential pathway to regulate P53 (Fig. 6A). GEPIA results showed that EXO1 expression was significantly correlated with PCNA, and CDC25C, which are important components of the P53 signaling pathway (Fig. 6B). Additionally, IHC of P53 in human tissues also demonstrated that P53 levels were distinctly lower in cancerous than in paracancerous tissues (Fig. 6C). To investigate the role of EXO1 in the regulation of P53 signaling, we examined the RNA, and protein levels of P53 after EXO1 knockdown. The results showed that EXO1 suppression led to a growth in P53 mRNA, and protein levels (Fig. 6D-E). Furthermore, we examined the expression of classical genes downstream of P53 including CDC25C and PCNA. CDC25C and PCNA expressions decreased visibly (Fig. 6F–I and Additional file 5: Fig. S5I). Conversely, when EXO1 was over-expressed, opposite changes were observed in these genes (Fig. 6J–O). Previous studies have shown that P53 signaling, when activated, promotes apoptosis by inhibiting BCL2 and promoting BAX; and inhibits the cell cycle by suppressing CDC25 and PCNA. To further confirm the effect of EXO1 on the P53 signaling pathway, we examined the expression of the P53 downstream signals BCL2, BAX, and CDC25C with PCNA after changing the expression level of EXO1. We found that EXO1 promoted BCL2, CDC25, and PCNA and inhibited BAX expression (Additional file 5: Fig. S5A–I), and further experiments showed that EXO1 inhibited apoptosis in prostate cancer cells. The above results suggest that EXO1 inhibits P53 signaling. In conclusion, these results supported that EXO1 inhibited P53 signaling.

### EXO1 regulated PCa lipid synthesis and progression through the P53 signaling

**Fig. 7:**
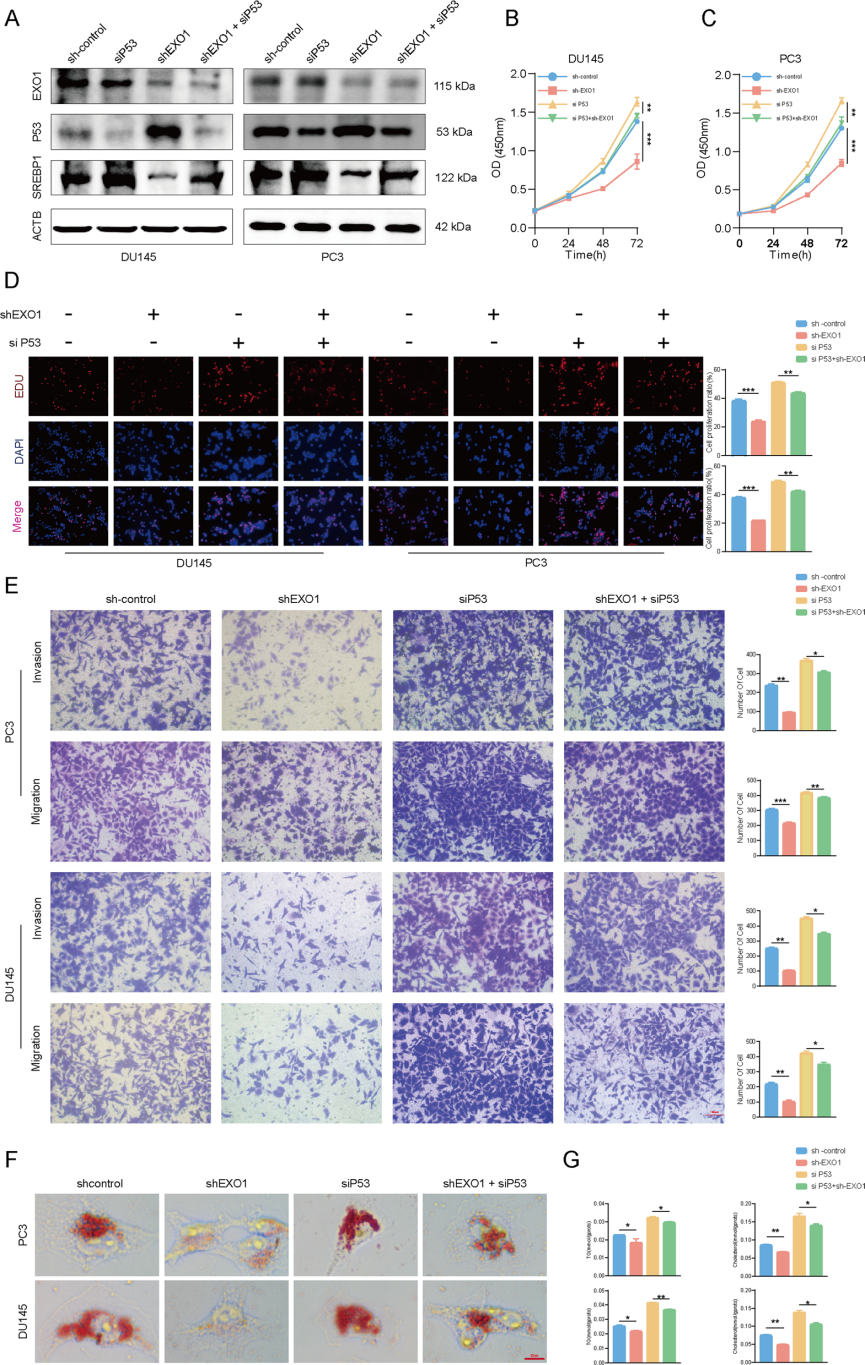
EXO1 promoted lipid synthesis and progression in prostate cancer by inhibiting P53 signaling. **A** In prostate cancer cells with knockdown of EXO1, P53 was further knocked down. EXO1, P53, and SREBP1 were detected using Western blot. **B**–**D** In the above cells, the proliferative ability of the cells was detected using CCK-8 and EdU assays. **E** In the above cells, the metastatic ability of the cells was determined us transwell assays. **F–G** In the above cells, lipid droplets were labeled using oil red O staining, and triglyceride, and cholesterol content was measured

To determine whether P53 mediated the EXO1 promotion of PCa progression and lipid synthesis, siRNA was used to inhibit the activity of P53 signaling after knocking down EXO1. The results of western blotting analysis suggested that P53 knockdown rescued the decreased expression of SREBP1 caused by EXO1 knockdown (Fig. 7A). Subsequently, many rescue experiments were conducted. The Results of CCK-8 and EdU assays showed that the proliferation ability of PCa cells was effectively rescued after P53 knockdown (Fig. 7B–D). Consistent with this, Transwell assays indicated that P53 knockdown rescued the metastasis ability of PCa cells (Fig. 7E). Additionally, oil Red O staining assays indicated that P53 knockdown effectively attenuated the lipid reduction induced by EXO1 knockdown (Fig. 7F). This finding was further validated by the results of TG, and cholesterol measurement experiments (Fig. 7G). Taken together, these results suggested that EXO1 promoted PCa progression by inhibiting P53 signaling. On this basis, we conclude that EXO1 promotes lipid synthesis and progression through PCa P53 signaling.

### EXO1 inhibited P53 signaling and promoted lipid synthesis in vivo

**Fig. 8:**
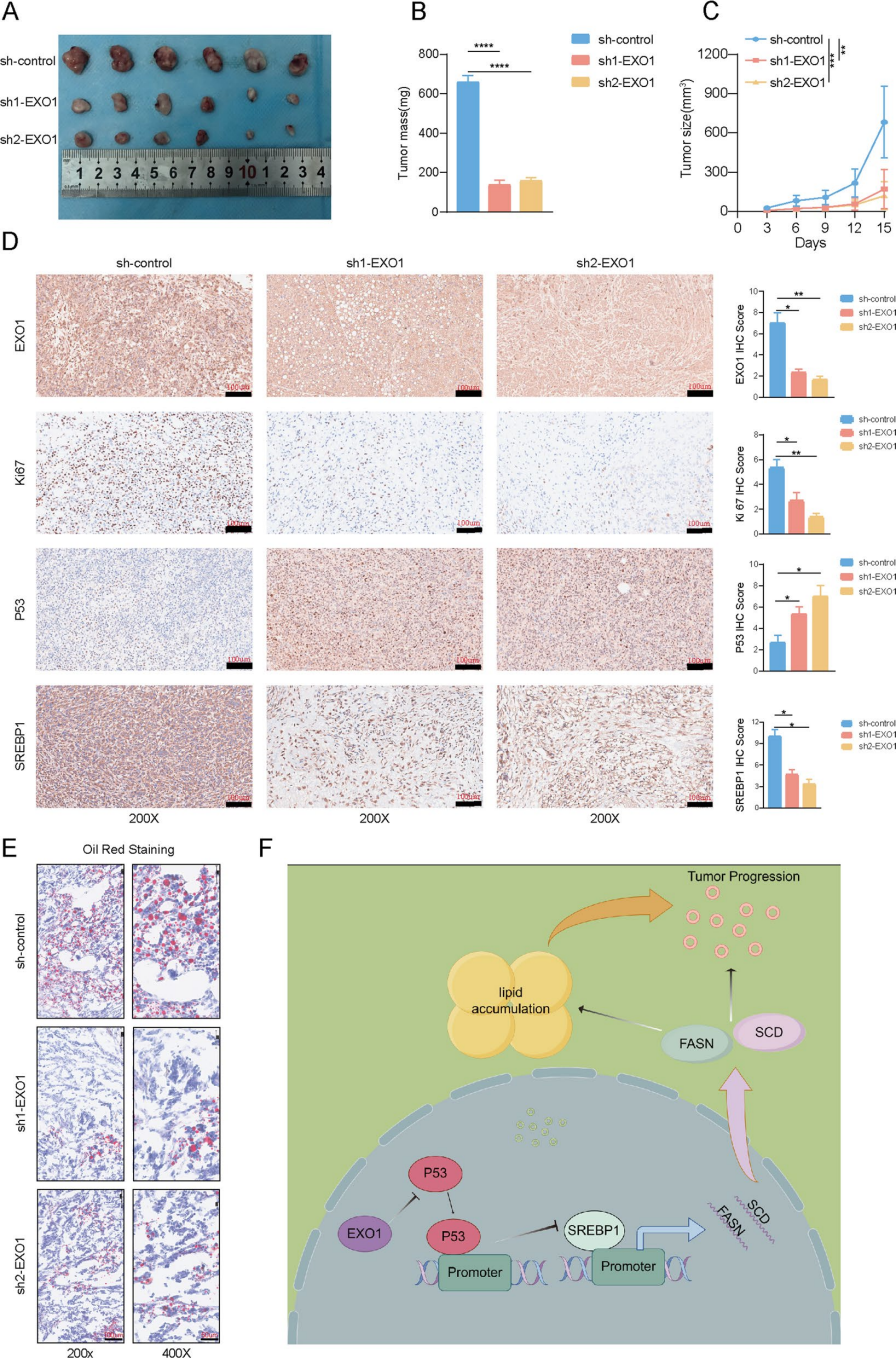
EXO1/P53/SREBP1 promoted prostate lipid synthesis and proliferation in vivo. **A, B** Photographs of the tumors in the control and EXO1 knocking down groups with weight statistics graphs (n = 6). **C** Tumor production curves **D** Immunohistochemistry to assess the expression levels of EXO1, P53, SREBP1, and Ki67 in the tumor tissues of the 3 groups. **E** Oil red staining of frozen sections of the 3 groups of tumors. **F** Graphical abstract briefly summarized EXO1/P53/SREBP regulation axis in the development of lipid metabolism in prostate cancer

Based on the results of the above in vitro experiments, we further examined the tumor growth-promoting and lipid accumulation effects of EXO1 in vivo. First, we constructed a PC3 cell line with stable knockdown of EXO1 using lentivirus and injected it subcutaneously in nude mice. The results showed that the tumor mass and volume were significantly reduced in the EXO1 knockdown group compared to the control group (Fig. 8A–C). Subsequently, subcutaneous tumors were used for IHC and oil red O staining. The results showed that the Ki67 scores remarkably decreased in the EXO1 knockdown group, suggesting that EXO1 significantly reduces the malignancy of PCa. The IHC results of EXO1, P53, and SREBP1 indicated that EXO1 inhibited P53 signaling, and promoted SREBP1 expression in vivo (Fig. 8D). Furthermore, the oil red O staining results showed that EXO1 knockdown significantly suppressed lipid accumulation in PCa (Fig. 8E). Collectively, our findings suggested that the EXO1-P53-SREBP1 axis promoted PCa progression and lipid accumulation, as observed in both in vitro and in vivo experiments (Fig. 8F).

## Discussion

Metastatic PCa (mPCa) is the leading cause of cancer-related death in men. The AR drives the progression of most mPCa, making androgen deprivation therapy (ADT) the first-line treatment option for mPCa. Although ADT and next-generation AR inhibitors initially improved disease burden, the patient response to this combination drug therapy varies widely because of molecular alterations in mPCa [34, 35]. In addition to altered AR signaling, the deletion of the potent oncogene p53 leads to adverse outcomes [36]. Therefore, new targets for PCa therapy related to P53 are needed. In our study, EXO1 was identified as a novel therapeutic target for PCa, and EXO1 inhibition activated P53 signaling, leading to the inhibition of PCa progression.

EXO1 is highly expressed in several cancers, including colorectal, breast, lung, prostate, and cervical cancers. Furthermore, it has been associated with poor prognosis in patients with tumors [7]. Our study suggested that EXO1 inhibited P53 signaling. This provides new insights into the mechanism of action of EXO1 in the prostate. TP53 mutations are strongly associated with PCa proliferation, and metastasis [37]. MDM2 and p53 proteins can activate, or suppress the p53 pathway in response to various stress signals [38]. The activation of P53 signaling regulates multiple transcriptional pathways, and its protein products are involved in several downstream cellular processes, including cell cycle, cell death, stem cell differentiation, cell metabolism, and DNA repair [39–41]. SREBP1 and SREBP2 are transcription factors involved in regulating the expression of genes involved in lipid metabolism. In particular, SREBP1 regulates the expressions of enzymes involved in fatty acid synthesis, whereas SREBP2 regulates enzymes involved in mevalonate synthesis. Previous studies have shown that P53 binds to the SREBP1 promoter region, and transcriptionally inhibits SREBP1 expression [14, 42]. Our results suggested that in PCa, P53 inhibited the mRNA, and protein levels of SREBP1, but had no effect on SREBP2 expression. This finding provides insights into the role of P53 in PCa development.

Metabolic abnormalities are one of the hallmarks of tumors. PCa cells exhibit increased lipid uptake and initial synthesis of fatty acids, and phospholipids, culminating in elevated lipid content [43, 44]. Obesity and a high-fat diet have been shown to promote PCa progression [45]. A Previous study showed that lipid storage alleviates endoplasmic reticulum stress, thereby promoting tumor progression [46]. Our results suggested that EXO1 promoted lipid synthesis, and lipid accumulation, and ultimately accelerated PCa progression by upregulating SREBP1 expression.

In conclusion, we found that EXO1 was a good biomarker for PCa by conducting a series of bioinformatic analyses including WGCNA. Furthermore, we clarified that EXO1 promoted PCa progression in vitro, and in vivo. Mechanistically, EXO1 promoted PCa lipid accumulation, and progression by inhibiting P53 signaling, and thereby promoting SREBP1 expression. Our study provides a potential target for the future treatment of PCa.

### Supplementary Information

**Additional file 1: Figure S1.** EXO1 was overexpressed in PCa. **A** The CNV distribution of EXO1 in PRAD tumor samples using the TCGA-PRAD project. **B** The Spearman correlation between EXO1 CNV and mRNA expression in TCGA-PRAD project. **C** The EXO1 methylation levels in TCGA-PRAD project. **D** The Spearman correlation between EXO1 methylation and mRNA expression in TCGA-PRAD project.

**Additional file 2: Figure S2.** Drug sensitivity analysis and immune infiltration analysis of EXO1. **A**–**D** Scatter plots were drawn using EXO1 expression as the horizontal coordinate and activity score of 6-thioguanine, Amonafide, Methylprednisolone, and Vorinostat as the vertical coordinate. **E** The activity scores for vorinostat in both high and low EXO1 expression groups. **F**–**H** Heatmap of EXO1 expression in tumors correlating with immune cells, immune activators, and immune checkpoints. **I** Scatter plots were drawn using the EXO1 expression as the horizontal and the activated CD4 expression as the vertical coordinate. **J** EXO1 expression correlates with molecular subtypes of immune subtypes.

**Additional file 3: Figure S3.** High concentrations of OA promoted PCa proliferation, migration, and invasion. **A**–**D** The proliferative capacity of cells after adding into BSA, 100 μm OA, and 200 μm OA was assayed by CCK-8, colony formation, and EdU assay.

**Additional file 4: Figure S4**. EXO1 promoted PCa proliferation by up-regulating SREBP1 for lipid synthesis. **A** In prostate cancer cells with knockdown of EXO1, SREBP1 was further overexpressed. EXO1 and SREBP1 were detected using Western blot. **B**-**C** In the above cells, the proliferative ability of the cells was detected using CCK-8. **D**–**G** In the above cells, triglyceride, and cholessterol content were measured. **H** In the above cells, the metastatic ability of the cells was determined using transwell assays.

**Additional file 5: Figure S5.** EXO1 regulated Molecules downstream of the P53 signaling pathway. **A**–**D** The mRNA expression of BCL2 in knocking down or overexpression of EXO1. **E**–**H** The mRNA expression of BCL2 in knocking down or overexpression of EXO1. **I** The WB analysis of P53, CDC25C, PCNA, BCL2, BAX of knocking down EXO1 or overexpressing EXO1.

**Additional file 6: Table S1.** The Detailed information of primer and antibody used in the experiment.

## Data Availability

The datasets and data used in this study can be obtained from the official website or corresponding author.

## Abbreviations

PCa: Prostate cancer
EXO1: Exonuclease 1
MMR: Mismatch repair
DSBR: Double strand break repair
HR: Homologous recombination
LOFs: Loss of functions
GOFs: Gain of functions
TCGA: The cancer genome atlas
GEO: Gene expression omnibus
UCSC Xena: University of California SANTA CRUZ Xena
DEG: Differentially expressed gene
WGCNA: Weighted gene co-expression network analysis
TOM: Topological overlap matrix
MEs: Module eigengenes
MM: Module membership
GS: Gene significance
ROC: Receiver operator characteristic
qRT-PCR: Reverse transcription-quantitative PCR
IHC: Immunohistochemistry
FBS: Fetal bovine serum
IBT: Immunoblotting test
PVDF: Polyvinylidene difluoride
ECL: Electrochemiluminescence
Ct: Threshold cycle
FDR: False discovery rate
GSEA: Gene set enrichment analysis
IHC: Immunohistochemistry
TBST: Ttis Buffered Saline Tween
KEGG: Kyoto Encyclopedia of Genes
GO: Gene Ontology
OS: Overall survival
DFS: Disease-free survival
RNA-seq: RNA sequencing
CCK-8: Cell Counting Kit-8
AUC: Areas under the curve
NES: Normalized enrichment score
BLAST: Basic Local Alignment Search Tool
SD: Standard Deviation
FASN: Fatty acid synthase
SCD: Stearoyl-Coa desaturase
OA: Oleic acid
TG: Triglyceride
T-CHO: Cholesterol
Edu assay: 5-Ethynyl-2’- deoxy uridine assay
K-M curve: Kaplan–Meier curve
AR: Androgen Receptors
ADT: Antiandrogen therapy
MDM2: MDM2 Proto-Oncogene
SREBP1: Sterol regulatory element binding transcription factor 1
