# Supplementary figures and images for: EXO1/P53/SREBP1 axis-regulated lipid metabolism promotes prostate cancer progression
Source: J Transl Med. 2024 Jan 26;22:104. doi: 10.1186/s12967-023-04822-z (PMC10811948; doi:10.1186/s12967-023-04822-z)

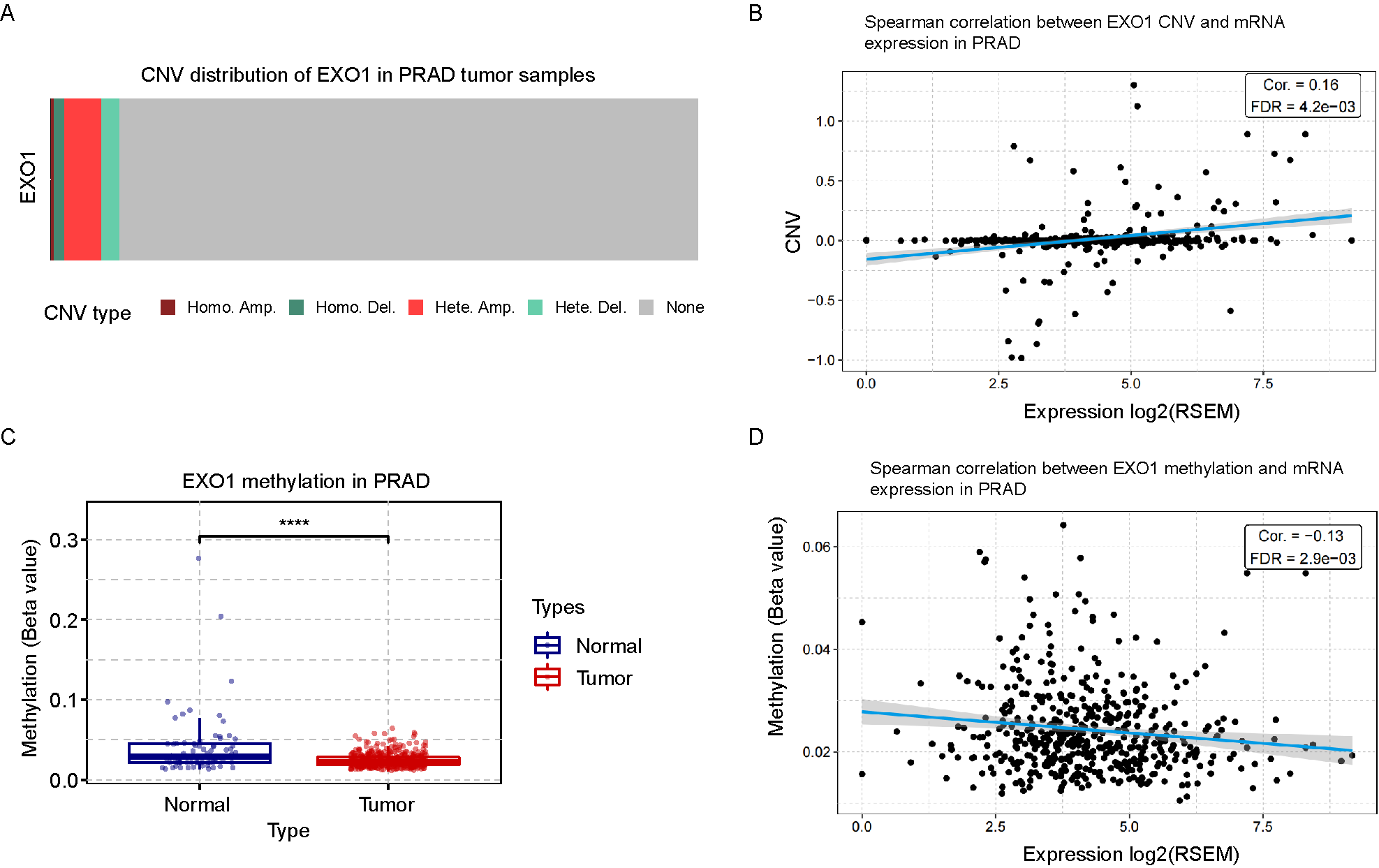

Supplement: Supplementary file 1 — Additional file 1: Figure S1. EXO1 was overexpressed in PCa. A The CNV distribution of EXO1 in PRAD tumor samples using the TCGA-PRAD project. B The Spearman correlation between EXO1 CNV and mRNA expression in TCGA-PRAD project. C The EXO1 methylation levels in TCGA-PRAD project. D The Spearman correlation between EXO1 methylation and mRNA expression in TCGA-PRAD project. [file 12967_2023_4822_MOESM1_ESM.tif]

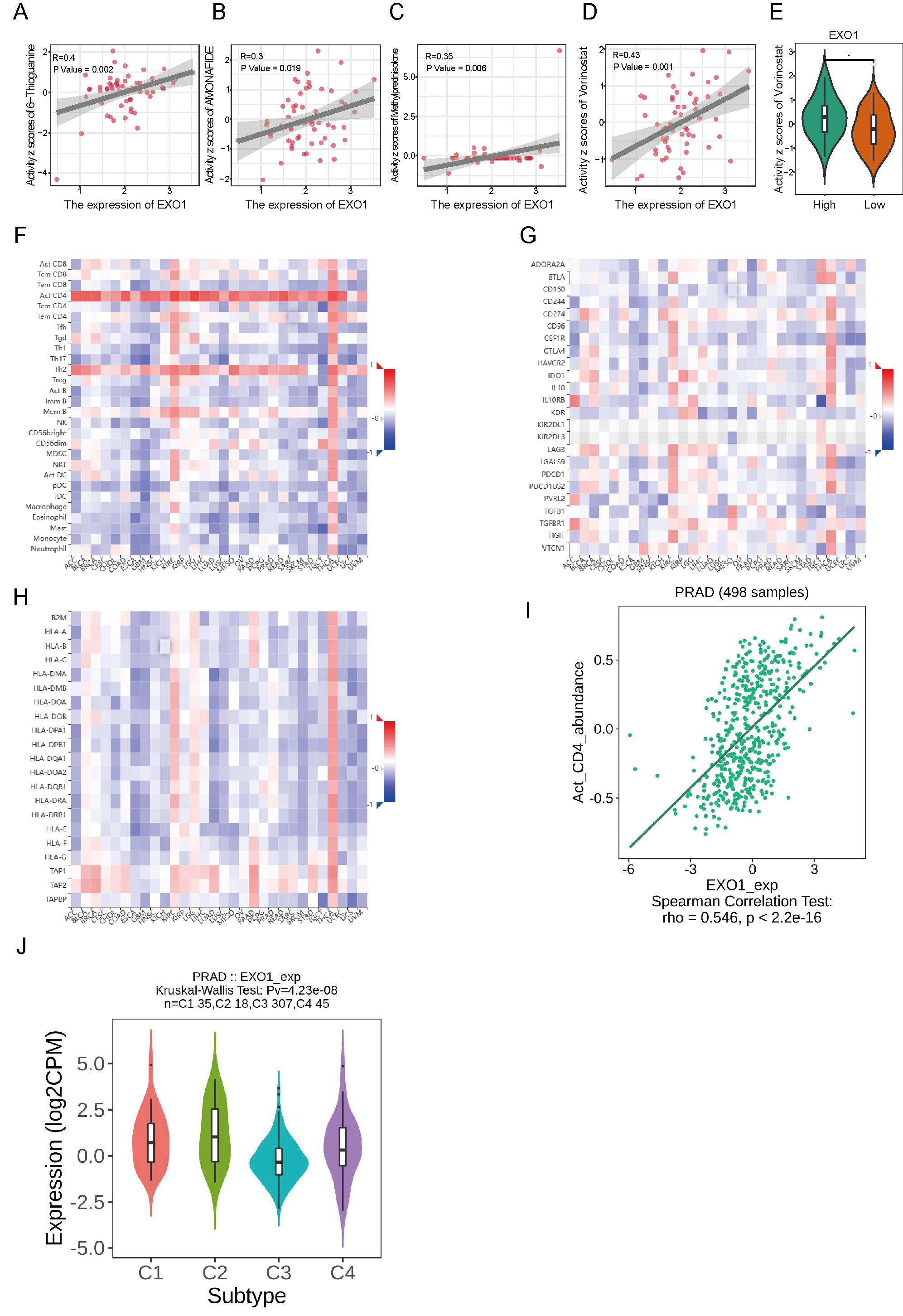

Supplement: Supplementary file 2 — Additional file 2: Figure S2. Drug sensitivity analysis and immune infiltration analysis of EXO1. A–D Scatter plots were drawn using EXO1 expression as the horizontal coordinate and activity score of 6-thioguanine, Amonafide, Methylprednisolone, and Vorinostat as the vertical coordinate. E The activity scores for vorinostat in both high and low EXO1 expression groups. F–H Heatmap of EXO1 expression in tumors correlating with immune cells, immune activators, and immune checkpoints. I Scatter plots were drawn using the EXO1 expression as the horizontal and the activated CD4 expression as the vertical coordinate. J EXO1 expression correlates with molecular subtypes of immune subtypes. [file 12967_2023_4822_MOESM2_ESM.tif]

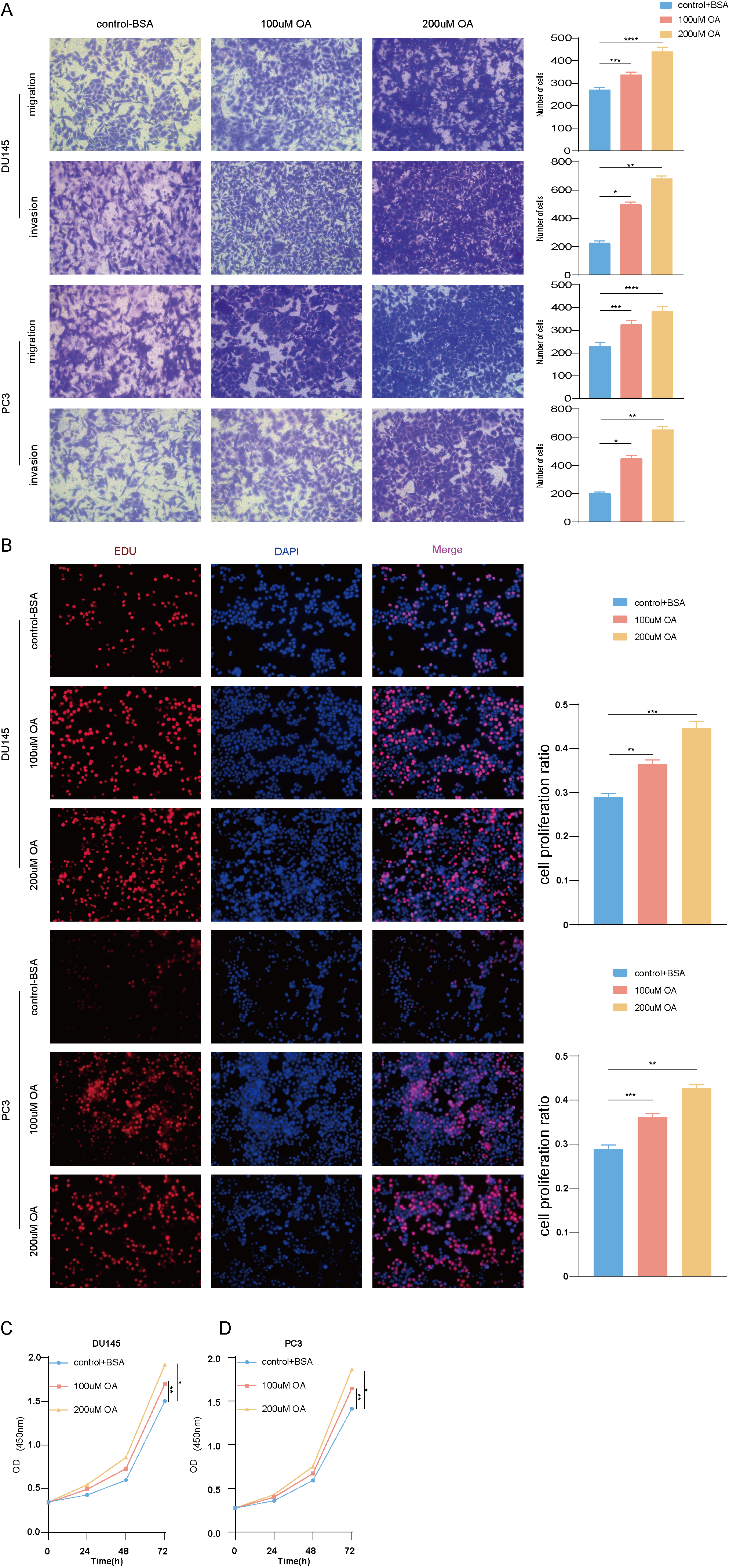

Supplement: Supplementary file 3 — Additional file 3: Figure S3. High concentrations of OA promoted PCa proliferation, migration, and invasion. A–D The proliferative capacity of cells after adding into BSA, 100 μm OA, and 200 μm OA was assayed by CCK-8, colony formation, and EdU assay. [file 12967_2023_4822_MOESM3_ESM.tif]

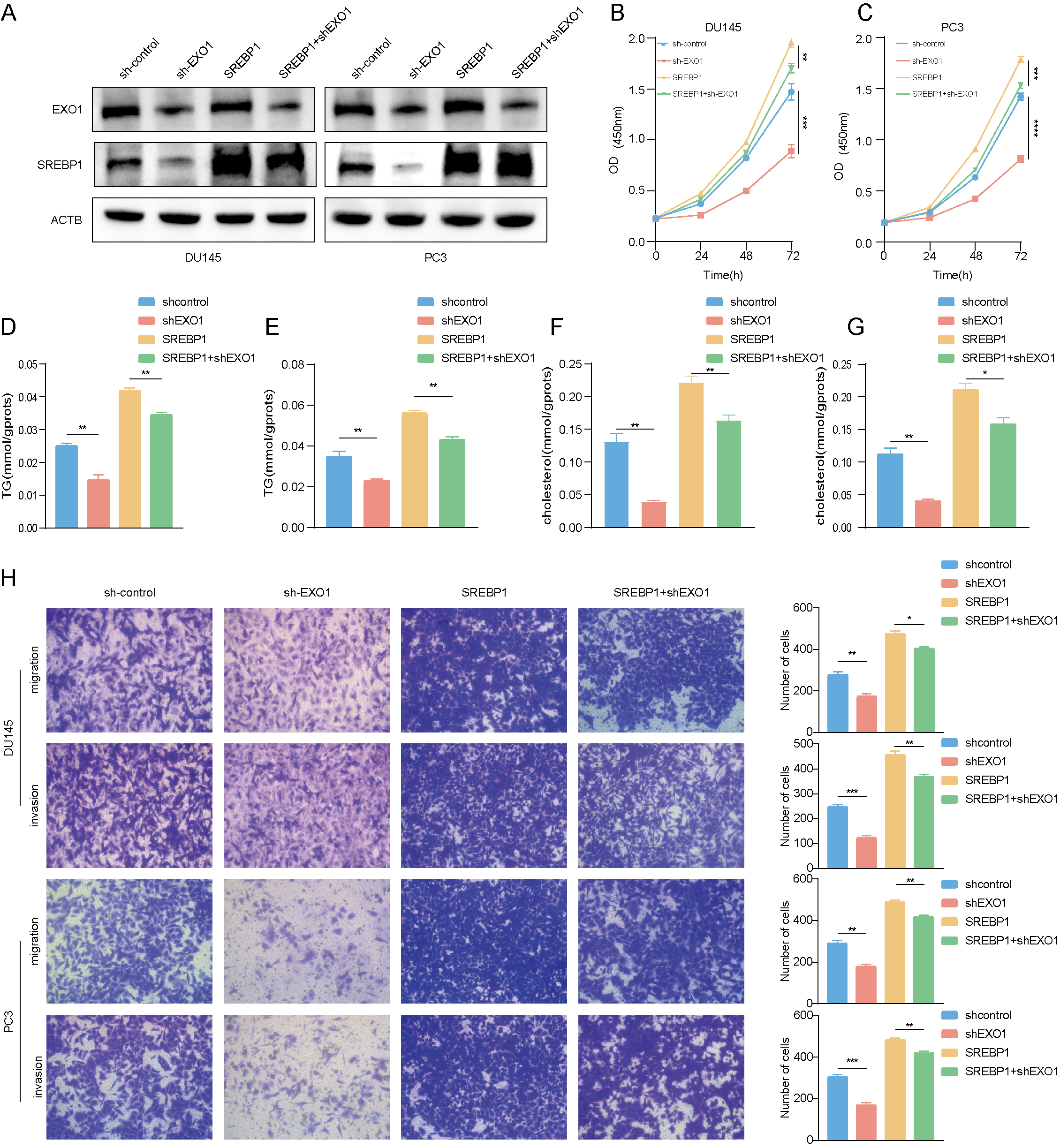

Supplement: Supplementary file 4 — Additional file 4: Figure S4. EXO1 promoted PCa proliferation by up-regulating SREBP1 for lipid synthesis. A In prostate cancer cells with knockdown of EXO1, SREBP1 was further overexpressed. EXO1 and SREBP1 were detected using Western blot. B-C In the above cells, the proliferative ability of the cells was detected using CCK-8. D–G In the above cells, triglyceride, and cholessterol content were measured. H In the above cells, the metastatic ability of the cells was determined using transwell assays. [file 12967_2023_4822_MOESM4_ESM.tif]

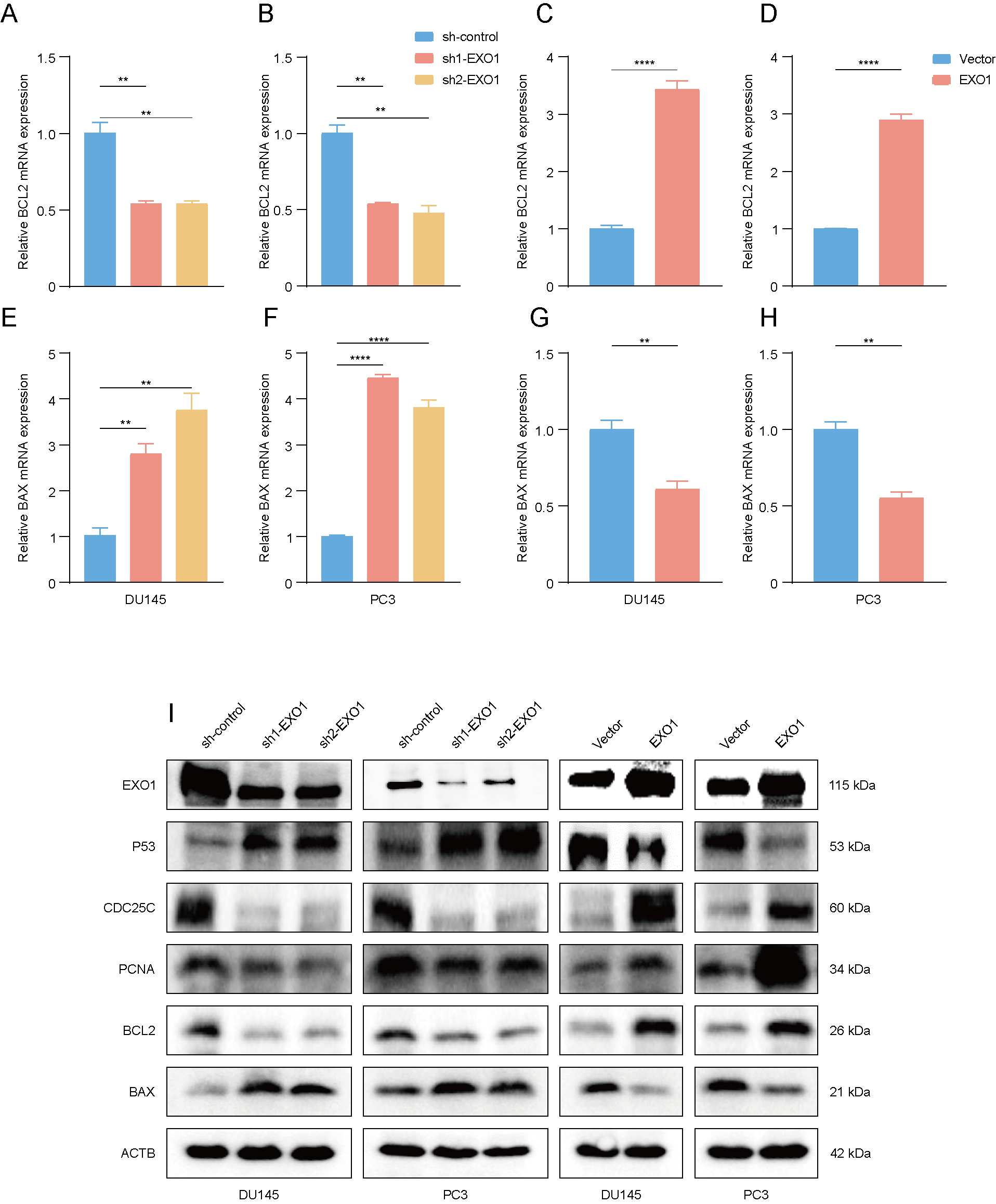

Supplement: Supplementary file 5 — Additional file 5: Figure S5. EXO1 regulated Molecules downstream of the P53 signaling pathway. A–D The mRNA expression of BCL2 in knocking down or overexpression of EXO1. E–H The mRNA expression of BCL2 in knocking down or overexpression of EXO1. I The WB analysis of P53, CDC25C, PCNA, BCL2, BAX of knocking down EXO1 or overexpressing EXO1. [file 12967_2023_4822_MOESM5_ESM.tif]
